# Role of activity and fibrosis in the MASLD atherogenic momentum through advanced lipidomics

**DOI:** 10.1371/journal.pone.0343134

**Published:** 2026-05-13

**Authors:** Agustín Blanco-Echevarría, Ramón Costa Segovia, Carlos Guijarro Herraiz, Belén García Izquierdo, Sonsoles Guadalix, Mercedes Pérez Carreras, Yolanda Rodríguez Gil, Marta de Castro Martínez, Nuria Amigó, Delia D’Avola, Carlos Lumbreras Bermejo, Diego Martínez-Urbistondo

**Affiliations:** 1 Unidad de Lípidos y Riesgo Vascular. Servicio de Medicina Interna. Hospital Universitario 12 de Octubre. Madrid, Spain; 2 Universidad Complutense de Madrid; 3 Instituto de Investigación Biomédica. Hospital Universitario 12 de Octubre “I+12”; 4 Unidad de Lípidos y Riesgo Vascular, Servicio de Medicina Interna, Fundación Hospital Alcorcón, Universidad Rey Juan Carlos, Madrid, Spain; 5 Área de Medicina Vascular, Departamento de Endocrinología, Clínica Universidad de Navarra, Madrid, Spain; 6 Servicio de Medicina Aparato Digestivo, Hospital Universitario 12 de Octubre, Universidad Complutense de Madrid, Madrid, Spain; 7 Servicio de Anatomía Patológica. Hospital Universitario 12 de Octubre; 8 Biosfer Teslab, Reus, Spain; 9 Department of Basic Medical Sciences, Rovira and Virgili University (URV), Tarragona, Spain; 10 Centre for Biomedical Research Network on Diabetes and Associated Metabolic Diseases (CIBERDEM), Instituto de Salud Carlos III, Madrid, Spain; 11 Unidad de Hepatología, Departamento de Medicina Interna, Clínica Universidad de Navarra, Madrid, Spain; 12 Área de Medicina Vascular, Departamento de Medicina Interna, Clínica Universidad de Navarra, Madrid, Spain; 13 Grupo PRESARV, Sociedad Española de Arteriosclerosis, Madrid, Spain; 14 CIBER de Fisiopatología de la Obesidad y Nutrición (CIBERobn), Instituto de Salud Carlos III, Madrid, Spain; University of Diyala College of Medicine, IRAQ

## Abstract

**Introduction:**

MASLD is linked to dyslipidemia, but it remains unclear whether this alteration depends more on disease activity or fibrosis.

**Objective:**

To determine whether dyslipidemia is primarily associated with activity and/or fibrosis through standard lipid profile and advanced lipidomics.

**Methods:**

A cross-sectional study was conducted including patients with suspected MASLD at a single center. Intraoperative liver biopsies were obtained. Only patients with MASLD were included in the analysis. Histological activity was defined as a NAFLD Activity Score (NAS) ≥5 and fibrosis was staged according to standard criteria. Clinical, biochemical, lipoprotein, and fatty acid profiles were assessed, including nuclear magnetic resonance (NMR) spectroscopy for lipoprotein subfractions and gas chromatography for fatty acids. Multivariable linear regression models were used to evaluate independent associations of activity and fibrosis with lipid parameters.

**Results:**

A total of 49 patients were analyzed (mean age 48 years, 46% men, 94% with BMI > 35). Liver biopsies revealed a 30.6% prevalence of activity (NAS ≥ 5) and 36.7% of fibrosis. Higher lipid levels in patients with activity were related to apolipoprotein B, non-HDL cholesterol, and triglycerides in both univariate and multivariate models. Fibrosis was not associated with any lipid parameter. Lipidomics confirmed associations of activity with VLDL+IDL cholesterol (β = 0.474, 95% CI 0.204–0.745, p = 0.001), VLDL+IDL triglycerides (β = 0.454, 95% CI 0.198–0.710, p = 0.001), and VLDL particle concentration (β = 0.409, 95% CI 0.155–0.663, p = 0.002), and showed a trend toward an inverse association with the percentage of large VLDL particles (β=−0.298, 95% CI −0.602–0.006, p = 0.054). Besides, the omega-6/triglycerides ratio was inversely associated with activity (β=−0.345, 95% CI −0.630 to −0.060, p = 0.019).

**Conclusion:**

Hepatic inflammatory activity showed strong associations with a non-traditional atherogenic lipidomic profile. These findings suggest that activity, rather than liver fibrosis, may be a key factor in the MASLD atherosclerotic momentum.

## Introduction

Metabolic dysfunction-associated steatotic liver disease (MASLD, formerly NAFLD) has emerged as a multisystem metabolic disorder affecting up to one-third of adults worldwide [1,2]. MASLD is often considered the hepatic manifestation of metabolic syndrome which includes central obesity, insulin resistance, atherogenic dyslipidemia, and hypertension [3]. Thus, cardiovascular disease (CVD) has become the leading cause of mortality in MASLD patients [4,5].

Notably, MASLD (especially more advanced forms) is associated with major adverse cardiovascular events (MACE) beyond traditional risk factors in observational liver biopsy-based cohorts [6] and meta-analyses [7,8]. These findings contrast with earlier large registry studies suggesting that MASLD does not independently increase cardiovascular risk after adjustment for conventional factors [9], leading to the exclusion of this condition from current cardiovascular risk assessment models [10]. This disparity highlights the need to clarify how MASLD is defined and measured from a metabolic perspective, in order to understand the contribution of this liver condition to cardiovascular risk [11,12].

The clinical definition of MASLD and its natural history—progressing from MASLD to MASH, MASH at risk, and eventually cirrhosis—appears well established, with fibrosis being the main predictor of hepatic decompensation and mortality [13]. However, inflammatory activity in MASH also plays a central pathophysiological role, acting as the driver of fibrogenesis [14]. A similar, though more complex, pattern has been described in the cardiovascular setting: liver disease progression is associated with increased cardiovascular events [6], but competing risks also arise, as some individuals develop cardiovascular disease before manifesting advanced liver disease [15]. In fact, Pennisi and colleagues demonstrated in a general population cohort that MASLD is linked to cardiovascular risk even in asymptomatic individuals, highlighting the bidirectional interplay between these conditions [16]. Proper methodological evaluation of both inflammatory activity and fibrogenesis processes is therefore needed, and assessing their relative contribution to atherosclerosis through lipid profile alterations might provide useful insight.

Lipid profiles are a cornerstone in predicting cardiovascular disease, yet traditional metrics (total cholesterol, LDL-C, HDL-C) have limitations in capturing residual lipid risk—a concept increasingly recognized in recent guidelines [17]. In translational terms, non-HDL cholesterol or measurement of apolipoprotein B provide a deeper initial insight into the atherogenic lipid burden [18]. Beyond that, advanced techniques such as NMR spectroscopy allow quantification of lipoprotein particle composition and size, enhancing prognostic value over conventional assays [19]. Similarly, analysis of the acylglyceride fraction within circulating triglycerides—specifically the relative proportions of omega-3, −6, −7, and −9 fatty acids—has demonstrated relevance for arterial wall biology and thus adds another layer to dyslipidemia assessment [20]. Assessing these alterations beyond traditional profiles in conditions such as MASLD activity versus fibrosis may help clarify the contribution of each pathological phase to cardiovascular risk.

The aim of this study was to explore whether MASLD inflammatory activity and fibrosis show different associations with advanced lipid parameters in high-risk patients, beyond traditional cardiovascular factors.

## Methods

### Study design and population

A cross-sectional study was conducted including consecutive patients undergoing liver biopsy due to MASLD suspicion at a single tertiary hospital in Spain between 10/07/2018 and 31/12/2021. Inclusion criteria were age ≥ 18 years, written informed consent for liver sampling and study participation, presence of steatosis in the liver biopsy, and adequate specimen quality to allow histopathological assessment of hepatic activity and fibrosis stage. Patients with secondary causes of steatosis, including any alcohol consumption, viral hepatitis, autoimmune liver disease, or use of hepatotoxic drugs, were not included if declared at baseline and were excluded if detected prospectively. All patients were included after signing informed consent. The study protocol was approved by the Institutional Review Board of Hospital Universitario 12 de Octubre (Madrid, Spain), under project number LIPID-RMN-18/ ABE-COL-2018-01.

### Clinical and biochemical assessment

Baseline demographic data (age and sex) and anthropometric measures, including body mass index (BMI), were recorded at the time of surgery. Clinical history was reviewed for the presence of metabolic syndrome components, including arterial hypertension, hypertriglyceridemia, low HDL cholesterol, and impaired fasting glucose. Use of lipid-lowering or antidiabetic medications was also registered. Fasting blood samples were obtained during the preoperative evaluation to determine standard biochemical parameters, including total cholesterol, LDL cholesterol, HDL cholesterol, non-HDL cholesterol, triglycerides, apolipoprotein B, and lipoprotein(a). Measurements were performed in serum samples using automated enzymatic and immunoturbidimetric assays in the hospital central laboratory, accredited according to international quality standards.

### Histological assessment

Liver biopsies were obtained by standard methods and processed according to standard protocols. Samples were fixed in formalin, embedded in paraffin, and stained with hematoxylin–eosin, Masson’s trichrome, and reticulin for histopathological evaluation. Steatosis, lobular inflammation, and hepatocellular ballooning were scored according to the NAFLD Activity Score (NAS). Significant activity was defined as NAS ≥ 5. Fibrosis was staged from F0 to F4 following the NASH Clinical Research Network criteria: F0, no fibrosis; F1, perisinusoidal or periportal fibrosis; F2, combined perisinusoidal and portal/periportal fibrosis; F3, bridging fibrosis; and F4, cirrhosis. Patients were classified as having fibrosis if any stage greater than zero was present. Biopsies with insufficient tissue or poor quality were excluded from the analysis. All samples were evaluated by experienced hepatopathologists which were blinded to the clinical and biochemical data.

### Lipoprotein and fatty acid assessment

Lipoprotein subfractions were analyzed by proton nuclear magnetic resonance (¹H-NMR) spectroscopy using a validated clinical platform (Biosfer Teslab, Reus, Spain). This method is based on the detection of lipid methyl group signals, which differ according to lipoprotein size and density, allowing direct quantification of lipoprotein particle number and lipid content across VLDL, IDL, LDL, and HDL subclasses. The methodology has been extensively validated and shows good agreement with ultracentrifugation-based reference methods [21] and potential clinical impact [22]. All spectra were processed with standardized algorithms, and quality control procedures were applied to ensure reproducibility

Serum fatty acids were analyzed in fasting samples using gas chromatography coupled with flame ionization detection (GC-FID) after lipid extraction and methylation, following established methods [23,24]. Fatty acid methyl esters were identified by comparison with certified reference standards and quantified as absolute concentrations (mg/dL) using internal standards and calibration curves. Quality control samples were included in each analytical batch to ensure analytical accuracy and reproducibility. The analysis included omega-3, omega-6, omega-7, and omega-9 fatty acids. Ratios of fatty acids to triglycerides were calculated to normalize fatty acid abundance relative to circulating triglyceride levels. Quality controls and calibration standards were included in each batch to ensure accuracy and reproducibility of measurements.

### Statistical analysis

Continuous variables are presented as medians and interquartile ranges (IQR). Comparisons between groups defined by activity (NAS < 5 vs. NAS ≥ 5) and by fibrosis stage (absence vs. presence) were performed using the Mann–Whitney U test. Categorical variables were expressed as absolute frequencies and percentages and compared using the chi-square test or Fisher’s exact test when appropriate. Multivariable analyses were conducted with linear regression models to evaluate the independent associations of activity and fibrosis with lipid, lipoprotein, and fatty acid parameters. Regression coefficients (β) with 95% confidence intervals (CI) and *p*-values are reported. All analyses were performed using SPSS Statistics, version 20 (IBM Corp., Armonk, NY, USA), and a two-sided *p* < 0.05 was considered statistically significant.

## Results

**Fig 1:**
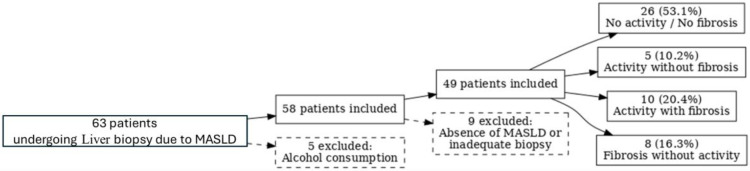
Study patients flowchart.

A total of 63 patients who underwent liver biopsy due to MASLD suspicion were initially considered. After excluding 5 individuals due to alcohol consumption, 58 remained eligible. Of these, 9 were excluded because of absence of MASLD or inadequate liver biopsy quality, resulting in 49 patients included in the final analysis. The study population included 49 adults with a mean age of 48 years and 46% men. Assessment of liver status in this cohort showed that 26 individuals (53.1%) had neither activity nor fibrosis, 5 (10.2%) presented activity without fibrosis, 10 (20.4%) had both activity and fibrosis, and 8 (16.3%) had fibrosis without significant activity. Among the 18 patients with fibrosis (both with and without inflammatory activity, the majority presented early-stage disease, with 12 patients classified as F1–F2 (66.7%), while fewer individuals had advanced fibrosis (F3 in 4 patients and F4 in 2 patients) (Fig 1).

Median BMI was 44.3 (40,9–50.0) and 42 patients (85.7%) fulfilled criteria for metabolic syndrome, with frequent hypertension, hypertriglyceridemia, low HDL cholesterol, and impaired fasting glucose. Approximately one third of patients were receiving statins and 10% were on oral antidiabetic drugs. Clinical characteristics were stratified by MASLD activity (NAS < 5 vs. NAS ≥ 5) and by the presence or absence of fibrosis. When patients were stratified by activity (NAS < 5 vs. NAS ≥ 5), those with higher activity showed significantly elevated total cholesterol (195.1 vs. 164.8 mg/dL, p = 0.013), non-HDL cholesterol (159.1 vs. 128.0 mg/dL, p = 0.017), apolipoprotein B (109.3 vs. 88.0 mg/dL, p = 0.013), and triglycerides (217.2 vs. 132.3 mg/dL, p = 0.006). No significant differences were observed between groups classified according to the presence or absence of fibrosis (Table 1). The distribution of this variables across groups defined by liver activity and fibrosis (Act-/Fib-, Act + /Fib-, Act + /Fib + , Act-/Fib+) is also shown (S1 Fig). Given these patterns, potential interaction between histological activity and fibrosis was evaluated by introducing an interaction term (activity × fibrosis) into multivariable linear regression models.

**Table 1:** Population characteristics classified by MASLD activity degree accoding to NAS score and MASLD fibrosis.

Variable	MASL [NAS < 5] (n = 34)	MASH [NAS ≥ 5] (n = 15)	p-value	Fibrosis = 0 (n = 31)	Fibrosis >0 (n = 18)	p-value
Demographic characteristics						
Age (years)	47.0 (36.8–53.8)	52.0 (37.0–56.5)	0.428	44.0 (36.5–52.5)	53.5 (40.0–57.8)	0.112
Male sex (%)	14 (41.2%)	8 (53.3%)	0.538	13 (41.9%)	9 (50.0%)	0.767
Active smoker (%)	6 (17.6%)	3 (20.0%)	0.679	6 (19.4%)	3 (16.7%)	1.000
BMI (kg/m²)	45.3 (41.4–51.5)	43.0 (41.1–44.7)	0.106	45.2 (43.1–51.3)	42.1 (39.2–47.9)	**0.052**
Metabolic Syndrome						
Hypertension (%)	30 (88.2%)	10 (66.7%)	0.110	26 (83.9%)	14 (77.8%)	0.708
Triglycerides criterion (%)	13 (38.2%)	9 (60.0%)	0.217	13 (41.9%)	9 (50.0%)	0.767
Low HDL criterion (%)	31 (91.2%)	12 (80.0%)	0.353	29 (93.5%)	14 (77.8%)	0.175
Glucose criterion (%)	20 (58.8%)	10 (66.7%)	0.754	16 (51.6%)	14 (77.8%)	0.127
Metabolic score	4.0 (3.0–4.8)	4.0 (3.0–5.0)	0.857	4.0 (3.0–4.0)	4.0 (3.0–5.0)	0.503
Active treatment						
Statin use (%)	8 (23.5%)	4 (26.7%)	1.000	5 (16.1%)	7 (38.9%)	0.094
ADO use (%)	7 (20.6%)	7 (46.7%)	0.089	5 (16.1%)	9 (50.0%)	**0.020**
Lipidic Profile						
Total cholesterol (mg/dL)	164.8 (146.1–175.0)	195.1 (164.9–216.8)	**0.013**	166.9 (147.2–178.3)	172.5 (160.8–209.6)	0.149
LDL cholesterol (mg/dL)	98.0 (77.6–113.4)	106.7 (89.6–134.5)	0.143	102.2 (79.1–112.6)	98.0 (87.0–129.5)	0.345
HDL cholesterol (mg/dL)	34.7 (28.1–39.8)	35.6 (30.6–44.0)	0.565	35.6 (29.9–39.8)	32.9 (27.3–45.1)	0.893
Non-HDL cholesterol (mg/dL)	128.0 (106.6–140.9)	159.1 (126.8–177.7)	**0.017**	129.4 (107.0–145.2)	133.5 (118.0–171.8)	0.210
Lipoprotein(a) (mg/dL)	26.0 (9.0–49.3)	11.2 (4.2–23.5)	**0.028**	25.7 (8.4–60.3)	19.7 (4.7–30.9)	0.164
Apolipoprotein B (mg/dL)	88.0 (79.0–96.0)	109.3 (84.5–116.5)	**0.013**	91.5 (79.0–100.0)	88.0 (80.6–111.5)	0.518
Triglycerides (mg/dL)	132.3 (103.5–163.2)	217.2 (122.5–280.5)	**0.006**	142.2 (104.2–167.3)	152.6 (116.9–236.8)	0.133

**Fig 2:**
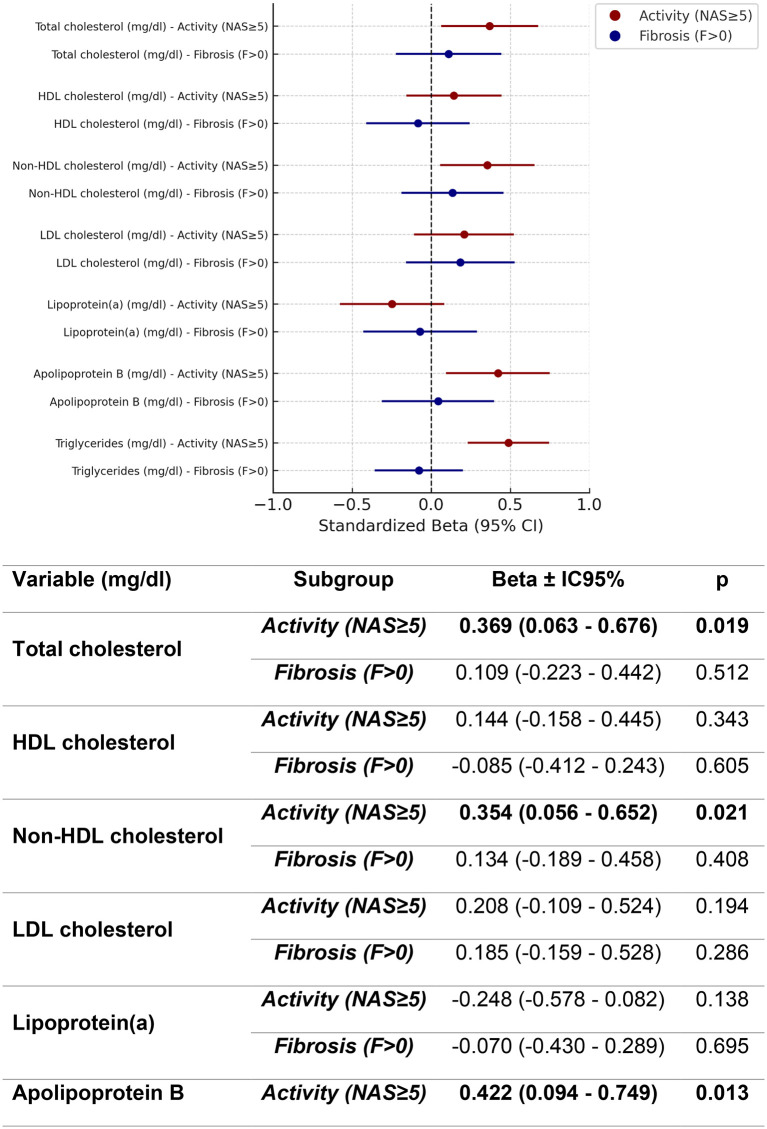
Multivariate regression analysis of the association of Activity and Fibrosis in MASLD to the traditional lipidic profile components adjusted by age, sex, metabolic score and metabolic treatment.

Therefore, both variables were subsequently considered simultaneously in multivariable models. In multivariable regression models including MASLD activity (NAS ≥ 5) and fibrosis (presence vs. absence), activity showed a significant association with total cholesterol (β = 0.369, 95% CI 0.063–0.676, p = 0.019), while fibrosis did not (β = 0.109, 95% CI −0.223 to 0.442, p = 0.512). Neither HDL nor LDL cholesterol demonstrated significant associations with either variable. Non-HDL cholesterol was related to activity (β = 0.354, 95% CI 0.056–0.652, p = 0.022) but not to fibrosis (β = 0.134, 95% CI −0.189 to 0.458, p = 0.408), and lipoprotein(a) remained unassociated in both comparisons. More consistent differences emerged for apolipoprotein B, which was linked to activity (β = 0.422, 95% CI 0.094–0.749, p = 0.014) but not to fibrosis (β = 0.043, 95% CI −0.312 to 0.399, p = 0.809), and for triglycerides, where activity showed a strong association (β = 0.489, 95% CI 0.236–0.742, p < 0.001) whereas fibrosis did not (β=−0.078, 95% CI −0.354 to 0.198, p = 0.576). Taken together, these models suggest that the significant signals in the association between activity and dyslipemia were not observed in the core lipid fractions captured by traditional risk scores (LDL and HDL cholesterol), but rather in non-HDL cholesterol, apolipoprotein B, and triglycerides (Fig 2).

Analysis of lipoprotein subfractions by NMR showed that activity (NAS ≥ 5) was associated with higher concentrations of VLDL cholesterol (28.4 vs. 17.0 mg/dL, p = 0.008), IDL cholesterol (11.3 vs. 9.6 mg/dL, p = 0.002), and VLDL triglycerides (117.1 vs. 77.1 mg/dL, p = 0.018), as well as an increased number of VLDL particles (92.4 vs. 56.6 nmol/L, p = 0.016) (Table 2).

**Table 2:** Apolipoprotein particle profiles analyzed in MRI spectrophotometry and classified by activity and fibrosis.

Variable	MASL [NAS < 5] (n = 34)	MASH [NAS ≥ 5] (n = 15)	p-value	Fibrosis = 0 (n = 31)	Fibrosis >0 (n = 18)	p-value
Cholesterol content of particles						
VLDL cholesterol (mg/dl)	17.0 (13.5–21.0)	28.4 (16.1–33.3)	**0.008**	16.9 (13.3–22.6)	20.3 (15.9–31.6)	0.073
IDL cholesterol (mg/dL)	9.6 (7.9–11.1)	11.3 (10.6–18.8)	**0.002**	10.1 (8.0–11.4)	10.6 (9.2–15.9)	0.149
LDL cholesterol (mg/dL)	127.5 (116.2–143.9)	145.5 (124.1–151.1)	0.155	129.4 (115.7–145.9)	134.2 (122.5–150.5)	0.345
HDL cholesterol (mg/dL)	51.2 (45.1–55.4)	45.1 (41.7–52.0)	0.264	49.7 (45.1–54.9)	49.3 (38.7–55.7)	0.487
Trygliceride content of particles						
VLDL triglycerides (mg/dL)	77.1 (58.7–96.7)	117.1 (74.1–169.7)	**0.018**	75.9 (62.1–106.4)	81.0 (63.1–144.0)	0.314
IDL triglycerides (mg/dL)	10.4 (9.0–11.4)	11.5 (11.0–16.1)	**0.007**	10.4 (9.2–11.9)	11.1 (10.3–12.9)	0.241
LDL triglycerides (mg/dL)	12.6 (11.0–16.5)	16.0 (13.4–18.0)	**0.029**	12.7 (11.2–16.4)	14.2 (12.6–18.3)	0.127
HDL triglycerides (mg/dL)	14.8 (12.6–17.4)	16.2 (13.3–18.9)	0.345	15.0 (12.6–17.7)	15.9 (12.2–17.5)	0.893
Number of particles						
VLDL particles (nmol/L)	56.6 (43.6–69.0)	92.4 (55.6–126.1)	**0.016**	55.7 (45.5–78.8)	63.0 (48.1–111.8)	0.258
LDL particles (nmol/L)	1291.0 (1207.2–1438.8)	1473.1 (1277.2–1606.7)	0.050	1304.7 (1190.0–1475.3)	1368.0 (1222.2–1592.2)	0.335
HDL particles (μmol/L)	25.3 (22.0–29.0)	25.6 (23.4–26.7)	0.905	25.8 (22.7–28.8)	24.2 (22.5–26.9)	0.514

**Fig 3:**
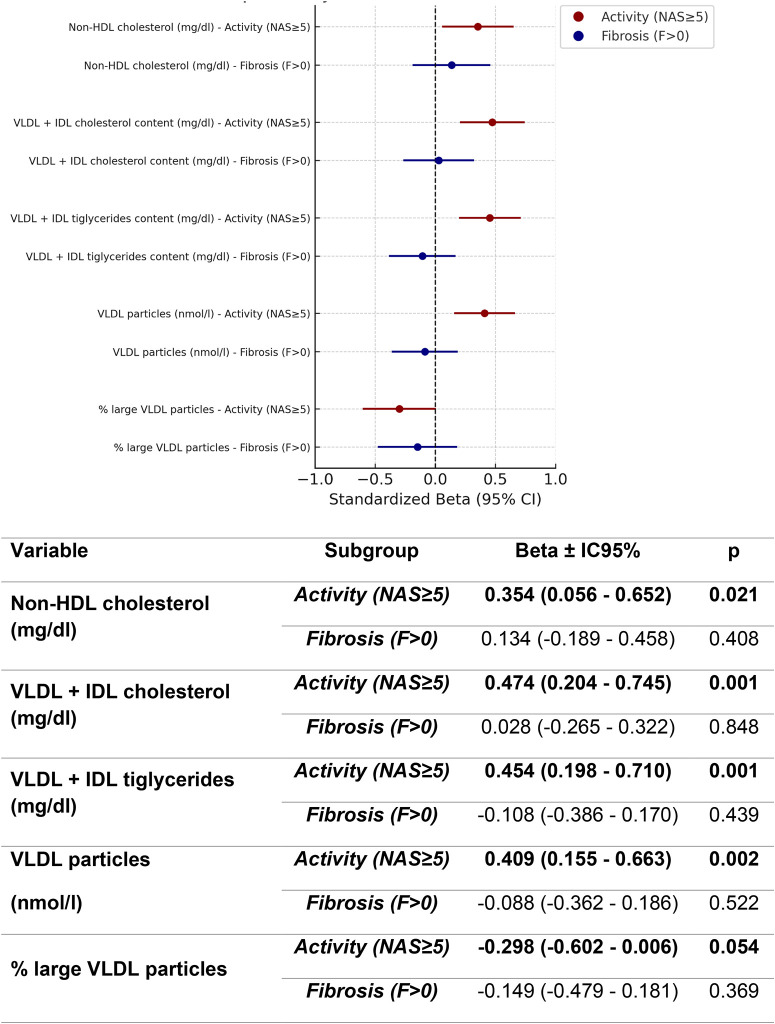
Multivariate regression analysis of the association of Activity and Fibrosis in MASLD to non-HDL components evaluated by MRI spectophotography adjusted by age, sex, metabolic score metabolic treatment and fibrosis.

In multivariable models including both activity and fibrosis, these findings were confirmed: activity was associated with combined VLDL+IDL cholesterol (β = 0.474, 95% CI 0.204–0.745, p = 0.001), VLDL+IDL triglycerides (β = 0.454, 95% CI 0.198–0.710, p = 0.001), and VLDL particle concentration (β = 0.409, 95% CI 0.155–0.663, p = 0.002), whereas fibrosis was not significantly related to any of these parameters. A trend toward an inverse relationship between activity and the proportion of large VLDL particles was also observed (β=−0.298, 95% CI −0.602 to 0.006, p = 0.054), without corresponding associations for fibrosis. No other lipoprotein subfractions showed significant associations with either variable (Fig 3).

Regarding acylglyceride profiles and omega fatty acids, patients with higher activity (NAS ≥ 5) showed elevated triglycerides (217.2 vs. 132.3 mg/dL, p = 0.006), omega-3 (0.304 vs. 0.241 mg/dL, p = 0.043), omega-9 (5.101 vs. 3.252 mg/dL, p = 0.020), while the omega-6/7 to triglycerides ratio was significantly lower (2.143% vs. 2.760%, p = 0.010) (Table 3).

**Table 3:** Acylglyceride profiles analyzed in MRI spectrophotometry and classified by activity and fibrosis.

Variable	MASLD [NAS < 5] (n = 34)	MASH [NAS ≥ 5] (n = 15)	p-value	Fibrosis = 0 (n = 31)	Fibrosis > 0 (n = 18)	p-value
Total concentration						
Triglycerides (mg/dL)	132.3 (103.4–163.1)	217.2 (122.4–280.5)	0.006	142.2 (104.2–167.2)	152.5 (116.8–236.8)	0.133
Omega-3 (mg/dL)	0.241 (0.205–0.290)	0.304 (0.237–0.373)	0.043	0.263 (0.227–0.296)	0.230 (0.198–0.335)	0.748
Omega-6/7 (mg/dL)	5.020 (4.453–5.629)	6.021 (4.425–6.750)	0.097	5.007 (4.260–5.892)	5.284 (4.528–6.107)	0.325
Omega-9 (mg/dL)	3.252 (2.846–3.925)	5.101 (3.079–6.576)	0.020	3.337 (2.873–4.166)	3.568 (2.912–5.273)	0.241
AG/Tryglicerides						
Omega-3 (% of Triglycerides)	0.127 (0.106–0.179)	0.131 (0.084–0.146)	0.334	0.131 (0.107–0.179)	0.126 (0.091–0.153)	0.325
Omega-6/7 (% of Triglycerides)	2.760 (2.344–3.449)	2.143 (1.667–2.682)	**0.010**	2.606 (2.310–3.207)	2.433 (1.857–2.923)	0.258
Omega-9 (% of Triglycerides)	1.771 (1.647–2.266)	1.805 (1.603–1.996)	0.428	1.767 (1.647–2.172)	1.863 (1.619–2.024)	0.554

**Fig 4:**
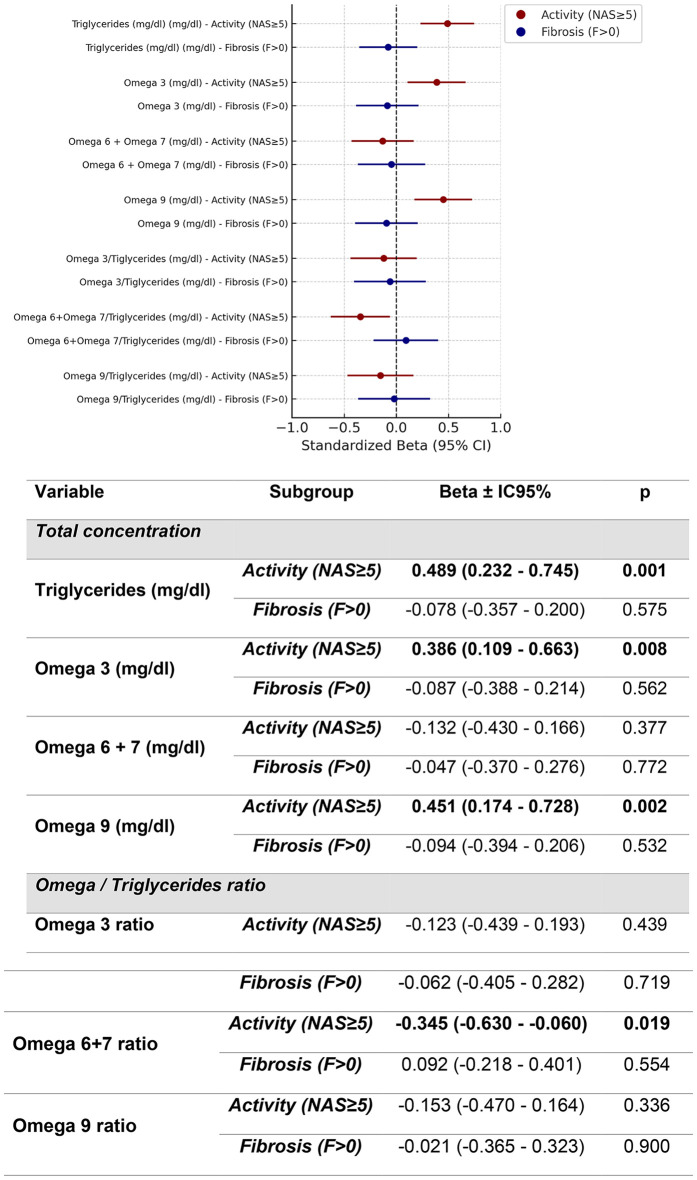
Multivariate regression analysis of the association of Activity and Fibrosis in MASLD to acylgliceride components evaluated by MRI spectophotography adjusted by age, sex, metabolic score metabolic treatment and fibrosis.

In multivariable models that simultaneously included activity and fibrosis, activity remained associated with triglycerides (β = 0.489, 95% CI 0.232–0.745, p = 0.001), omega-3 (β = 0.386, 95% CI 0.109–0.663, p = 0.008), and omega-9 (β = 0.451, 95% CI 0.174–0.728, p = 0.002), and was inversely associated with the omega-6/7 to triglycerides ratio (β=−0.345, 95% CI −0.630 to −0.060, p = 0.019). None of these variables showed significant associations with fibrosis (Fig 4).

Exploratory interaction analyses were performed to assess potential effect modification between histological activity (NAS ≥ 5) and the main components of the metabolic syndrome (arterial hypertension, impaired fasting glucose/diabetes, hypertriglyceridemia, and low HDL cholesterol). No statistically significant interaction terms were observed for any of the lipid or lipidomic parameters assessed.

## Discussion

**Fig 5:**
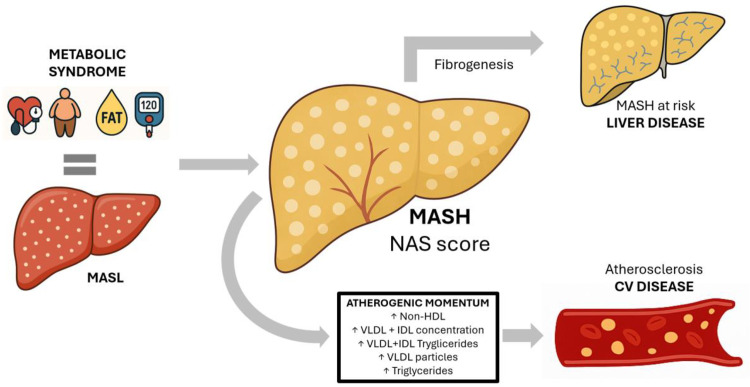
Inflammatory liver burden as lipidic disruptor beyond cardiovascular risk factors and fibrosis.

The findings of this biopsy-based MASLD cohort suggest that hepatic inflammatory activity (NAS) may represent the dominant driver of atherogenic dyslipidemia, independently of the degree of fibrosis. Beyond higher non-HDL cholesterol and apoB, the lipidomic profile in MASH was not merely quantitatively worse but compositionally enriched in VLDL/IDL fractions: VLDL-cholesterol +67%, IDL-cholesterol +18%, VLDL-triglycerides +52% and VLDL particle number +63%. All associations were independent of age, sex, metabolic score, lipid-lowering or antidiabetic therapy, and presence of liver fibrosis. Consistently, NAS related directly to circulating triglycerides (+64%) and inversely to the relative abundance of omega-6/7 fatty acids within the triglyceride pool (−22%), underscoring inflammatory burden as the principal metabolic disruptor beyond traditional cardiovascular risk factors and fibrosis (Fig 5).

These results align with emerging pathophysiological frameworks positioning hepatic inflammation—not fibrosis—as a key amplifier of atherogenic dyslipidemia in MASLD [25,26]. Experimental and translational studies indicate that heightened hepatocellular stress and immune activation in MASH stimulate de novo lipogenesis and VLDL overproduction via SREBP-1c and related lipogenic transcription factors, leading to increased secretion of large, triglyceride-rich VLDL particles and impaired peripheral clearance [27–29]. Notably, such metabolic derangements occur early in the disease process and may precede substantial fibrotic remodeling. The elevation of VLDL particle number observed in our cohort provides mechanistic plausibility, as this trait correlates with hepatic triglyceride flux and insulin resistance—both hallmarks of MASH [26]. Indeed, intrahepatic steatosis and active steatohepatitis in MASLD are associated with increased VLDL particle numbers and larger VLDL size [26], whereas fibrosis showed distinct effects on VLDL composition without a proportional increase in particle output [26].

Our study reinforces the view that cardiovascular disease (CVD) risk in MASLD is largely driven by inflammatory and metabolic perturbations rather than fibrosis burden. While fibrosis stage is a well-established prognostic marker for liver-related outcomes [4], the relationship of liver fibrosis with CVD risk is less clear [30]. Prior studies have yielded conflicting data: some report that advanced fibrosis correlates with higher incident cardiovascular events [31,32], whereas others—especially those accounting for metabolic syndrome—find minimal independent association between fibrosis and CVD [32]. Besides, composite scores linking hepatic dysfunction and systemic inflammation with cardiovascular outcomes have been proposed, although these markers are not incorporated into established cardiovascular risk models, and their role in individual cardiovascular risk stratification in MASLD remains to be defined [33,34]. Our data support the latter and suggest that NAS, as a dynamic indicator of hepatic inflammation and injury, better reflects the atherogenic impact of the liver on systemic metabolism. In contrast, in our cohort, fibrosis stage showed no independent association with lipid subclass patterns, which underscores the dominant role of histologic activity in driving the MASLD “atherogenic momentum.”

Importantly, one must differentiate this atherogenic momentum of active MASH from established atherosclerosis when considering fibrosis–CVD links. Both advanced fibrosis and atherosclerotic vascular disease might serve as independent markers of prolonged exposure to the MASH metabolic-inflammatory milieu, rather than fibrosis directly causing cardiovascular events [11]. This distinction is critical, as current cardiovascular risk models do not incorporate liver histology, and surrogate markers of hepatic inflammatory activity are scarce and costly. Emerging metabolomic panels (e.g. OWLiver® test) can noninvasively detect MASH [35], but their availability is limited and they are not yet validated for routine risk stratification.

Moreover, advanced lipidomics of plasma acylglycerides reinforces a pro-inflammatory milieu in MASH. As expected, patients with MASH carried a higher triglyceride burden (+64% vs MASLD without activity; 217 vs 132 mg/dL; adjusted β 0.489, p = 0.001). Yet omega-class acylglycerides linked to anti-inflammatory pathways were higher in absolute concentration (omega-6/7: 6.02 vs 5.02 mg/dL; + 20%, p = 0.097) but lower in relative terms when expressed as a proportion of the triglyceride pool (omega-6/7 ratio −22%; 2.14% vs 2.76% of TG; p = 0.010), and this inverse association with histologic activity persisted after multivariable adjustment (β −0.345 [−0.630 to −0.060]; p = 0.019). This may be mediated by upregulated fatty acid desaturase activity, oxidative stress and impaired peroxisomal fatty acid oxidation in an inflamed liver [36,37]. Whether these changes are a cause or consequence of hepatic inflammation remains uncertain, but they provide a strong rationale for exploring dietary or pharmacological interventions targeting not just lipid quantity but also lipid quality in MASLD.

From a clinical perspective, these findings underscore that MASLD is not merely a liver ailment but a systemic metabolic condition with major cardiovascular implications. In this hypothesis-generating cohort, non-HDL cholesterol, apolipoprotein B, and triglycerides emerged as the lipid parameters most consistently associated with hepatic inflammatory activity, while fibrosis showed no independent association. When available, VLDL particle concentration further reflected this activity-related atherogenic pattern. Although these findings may help frame future research on lipid-based stratification of atherosclerotic risk in MASLD, they should not be interpreted as defining a standardized or validated clinical profile and require confirmation in larger, prospective studies. Acccording to our findings, strategies may prioritize resolution of hepatic inflammation and metabolic dysfunction alongside traditional risk factor control. Several agents targeting hepatic steatosis and inflammation—such as GLP-1 receptor and dual GLP-GIP receptor agonists—have shown promise in improving cardiovascular risk markers in MASLD, independent of fibrosis regression [38,39]. Likewise, resmetirom markedly decreases atherogenic lipids (mean ~24% reduction in LDL-C and ~34% reduction in triglycerides) in NASH patients, an effect achieved even before any fibrosis improvement, and without excess adverse events [40]. Our data suggest that patients with high NAS but low fibrosis may represent a high-risk, early-intervention subgroup that could benefit the most from such approaches aimed at mitigating the “metabolic storm” of MASLD.

Finally, while our study is strengthened by liver biopsy confirmation and advanced lipoprotein profiling, it has limitations. The cross-sectional design and small sample limits causal inference, and the modest sample size may reduce power to detect subtle associations, especially in fibrosis subgroup analyses. In addition, fibrosis was predominantly incipient in this cohort (F1-F2 12 of 18 patients with fibrosis, 66.7%), which precluded statistically robust stratification across individual fibrosis stages; however, this does not detract from the proof-of-concept nature of the study, aimed at exploring differential lipidomic signatures associated with hepatic inflammatory activity versus fibrosis. Conversely, the fact that the cohort consisted of individuals with very high metabolic risk—a feature previously used to define MASLD phenotypes—enhances the robustness and relevance of our findings [11]. Furthermore, we did not assess dietary intake, genetic polymorphisms (e.g., PNPLA3, TM6SF2) or gut microbiome composition, all of which could influence lipid metabolism and disease activity. Exploratory interaction analyses between histological activity and components of the metabolic syndrome were limited by sample size and may have been underpowered to detect modest interaction effects. For these reasons, this study is explicitly hypothesis-generating. These data suggest that hepatic inflammatory activity and its lipidomic signature may aid patient stratification to guide therapy and support further validation in prospective studies with cardiovascular outcomes.

## Conclusion

Hepatic inflammatory activity — not fibrosis — appears to be the primary driver of an atherogenic lipoprotein profile in MASLD. This potential paradigm shift calls for reframing MASLD as a metabolically active organ disease with systemic sequelae and supports therapeutic targeting of hepatic inflammation to reduce cardiovascular risk in this population.

## Supporting information

S1 Fig Distribution of atherogenic lipid parameters according to NAS and fibrosis status. Non-HDL cholesterol, apolipoprotein B, and triglyceride levels across subgroups defined by NAS (NAS + vs NAS−) and fibrosis status (Fib + vs Fib−).
(PDF)
