# Supplementary material for: Role of activity and fibrosis in the MASLD atherogenic momentum through advanced lipidomics
Source: PLoS One. 2026 May 13;21(5):e0343134. doi: 10.1371/journal.pone.0343134 (PMC13170956; doi:10.1371/journal.pone.0343134)

Supplementary Figure 1. Specific standard lipidic analysis as distributed in MASLD status subgroups according to activity and fibrosis.

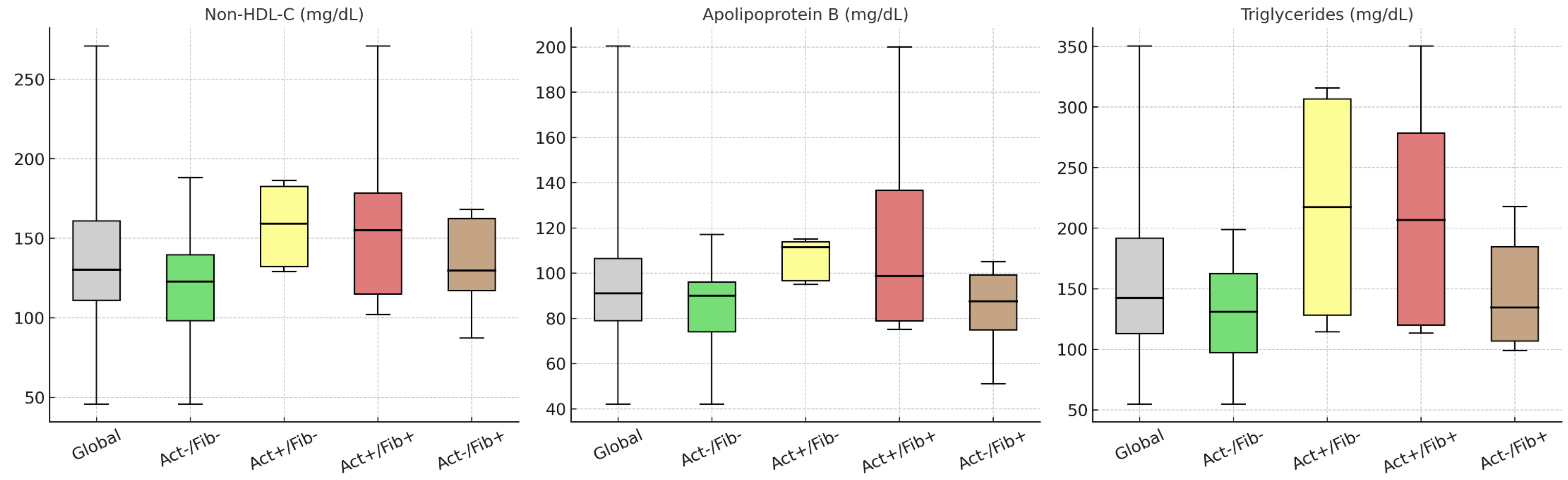

Supplement: S1 Fig — Non-HDL cholesterol, apolipoprotein B, and triglyceride levels across subgroups defined by NAS (NAS + vs NAS−) and fibrosis status (Fib + vs Fib−). (PDF) [file pone.0343134.s001.pdf]
